# Photon Counting CT and Radiomic Analysis Enables Differentiation of Tumors Based on Lymphocyte Burden

**DOI:** 10.3390/tomography8020061

**Published:** 2022-03-10

**Authors:** Alex J. Allphin, Yvonne M. Mowery, Kyle J. Lafata, Darin P. Clark, Alex M. Bassil, Rico Castillo, Diana Odhiambo, Matthew D. Holbrook, Ketan B. Ghaghada, Cristian T. Badea

**Affiliations:** 1 Quantitative Imaging and Analysis Lab, Department of Radiology, Duke University Medical Center, Durham, NC 277101, USA; darin.clark@duke.edu (D.P.C.); matthew.holbrook@duke.edu (M.D.H.); 2 Department of Radiation Oncology, Duke University Medical Center, Durham, NC 27710, USA; yvonne.mowery@duke.edu (Y.M.M.); kyle.lafata@duke.edu (K.J.L.); alex.bassil@duke.edu (A.M.B.); ricojames.castillo@duke.edu (R.C.); diana.odhiambo@duke.edu (D.O.); 3 Department of Head and Neck Surgery & Communication Sciences, Duke University Medical Center, Durham, NC 27710, USA; 4 Department of Radiology, Duke University, Durham, NC 27710, USA; 5 Department of Electrical and Computer Engineering, Duke University, Durham, NC 27710, USA; 6 E.B. Singleton Department of Radiology, Texas Children’s Hospital, Houston, TX 77030, USA; ghaghada@bcm.edu; 7 Department of Radiology, Baylor College of Medicine, Houston, TX 77030, USA

**Keywords:** micro-CT, spectral CT, photon counting detector, preclinical, nanoparticles, radiomics, radiogenomics

## Abstract

The purpose of this study was to investigate if radiomic analysis based on spectral micro-CT with nanoparticle contrast-enhancement can differentiate tumors based on lymphocyte burden. High mutational load transplant soft tissue sarcomas were initiated in *Rag2*^+/−^ and *Rag2*^−/−^ mice to model varying lymphocyte burden. Mice received radiation therapy (20 Gy) to the tumor-bearing hind limb and were injected with a liposomal iodinated contrast agent. Five days later, animals underwent conventional micro-CT imaging using an energy integrating detector (EID) and spectral micro-CT imaging using a photon-counting detector (PCD). Tumor volumes and iodine uptakes were measured. The radiomic features (RF) were grouped into feature-spaces corresponding to EID, PCD, and spectral decomposition images. The RFs were ranked to reduce redundancy and increase relevance based on TL burden. A stratified repeated cross validation strategy was used to assess separation using a logistic regression classifier. Tumor iodine concentration was the only significantly different conventional tumor metric between *Rag2*^+/−^ (TLs present) and *Rag2*^−/−^ (TL-deficient) tumors. The RFs further enabled differentiation between *Rag2*^+/−^ and *Rag2*^−/−^ tumors. The PCD-derived RFs provided the highest accuracy (0.68) followed by decomposition-derived RFs (0.60) and the EID-derived RFs (0.58). Such non-invasive approaches could aid in tumor stratification for cancer therapy studies.

## 1. Introduction

The immune response to neoplastic disease involves infiltration by lymphocytes (including T cells and B cells) [1]. T cells and B cells are the major cellular components of the adaptive immune response, and they play an important role in cancer progression and response to certain therapeutics, particularly to immunotherapy [2]. Assessment of tumor lymphocytes (TLs) is usually performed by histopathology and may provide important prognostic information in tumors [3]. However, implementation as a routine clinical biomarker has not yet been achieved. Furthermore, biopsy-based methods are limited due to both patient discomfort and risk with repeated invasive procedures, as well as sampling error due to tumor heterogeneity and the small volume of tissue obtained with each biopsy. Advanced quantitative imaging-based methods that enable non-invasive and repeated whole-tumor interrogation are highly desirable. A recent study presents such an attempt based on PET imaging [4]. However, we are not aware of any CT imaging of TL burden. We focus on CT because it is one of the most commonly used modalities in cancer imaging. We hypothesize that high-resolution and spectral CT imaging can provide rich datasets required for radiomics assessment of lymphocytic presence in tumors.

Radiomics is a rapidly growing field in diagnostic radiology, wherein images are data mined to define phenotypic characteristics of tumors by extracting quantitative image features such as intensity, shape, size, morphology, and texture [5]. The utility of radiomics benefits greatly from the use of machine learning algorithms [6]. Supervised and unsupervised data-driven machine learning approaches have shown promise in various aspects of medical image analysis. These solutions offer the potential to assist radiologists in therapy [7], disease classification [8], and prognosis pre-evaluation [9]. Machine learning can be applied directly to images or can be trained on data derived from radiomic analysis. We hypothesize that radiomic analysis may offer a rich, standardized corpus of data that can be used with such approaches independent of image modality or device manufacturer.

Furthermore, radiomics provides an innovative approach for identifying and developing quantitative cancer imaging biomarkers that are otherwise not possible when based on conventional imaging-based metrics. Spectral information obtained using photon-counting detectors (PCD) can improve radiomic analysis based on CT. PCD CT can generate images with less noise, higher resolution, and improved contrast-to-noise ratio when compared to conventional CT based on energy integrating detectors (EID) [10]. Our group has developed PCD-based micro-CT prototypes [11,12] for quantitative cancer imaging and for testing novel nanoparticle (NP) contrast agents [13,14,15] for spectral CT imaging. NPs accumulate in tumors due to the enhanced permeability and retention (EPR) effect [16,17]. Furthermore, NPs modified with various targeting strategies can facilitate molecular imaging with spectral PCD CT [18]. 

In this work, we performed a preclinical study using NP contrast-enhanced imaging followed by radiomic analysis on both conventional EID and spectral PCD-based micro-CT images to investigate differentiation of sarcomas based on TLs using *Rag2*^−/−^ (TL-deficient) and littermate control *Rag2*^+/−^ (TLs present) mice [19]. A similar radiomics study investigating differences associated with tumor-associated macrophages was recently published [20]; however, in that work, the authors used conventional single energy micro-CT imaging. We have thus explored the possible comparative benefits of using spectral PCD versus conventional EID CT data within the realm of radiomic analysis.

## 2. Materials and Methods

**Figure 1:**
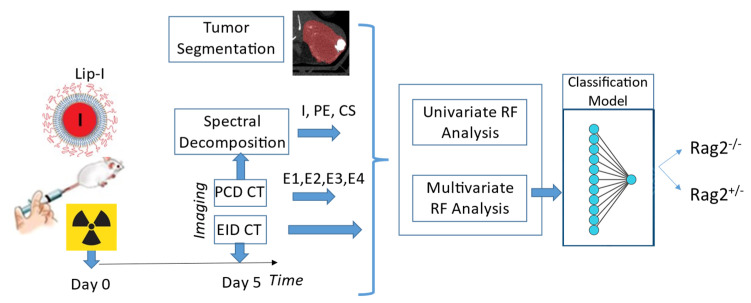
The timeline of our experiments and the analysis involved in our study.

Figure 1 presents the experimental timeline and major steps of the analysis. Mice received radiation therapy (RT) to induce lymphocytic infiltrates and were injected immediately after with a liposomal iodine (Lip-I) NP contrast agent. Five days later, animals underwent delayed NP contrast-enhanced CT imaging. Image analysis was performed on EID-CT, PCD-CT (4 energies, $E_{i}=1\ldots 4$) and derived spectral decomposition maps of Iodine (I), Photoelectric effect (PE) and Compton Scattering (CS) images, separately. Radiomic features were used to train a classifier that separates between the two types of mice. We describe each step of our study in the following subsections.

### 2.1. Mouse Models

All animal procedures were approved by Duke University Institutional Care and Use Committee (IACUC) and adhered to the NIH Guide for the Care and Use of Laboratory Animals. A high mutational load tumor cell line was generated from an autochthonous soft tissue sarcoma (p53/MCA model [19,21]) induced in a C57BL/6 wild type mouse by intramuscular injection of adenovirus expressing Cas9 endonuclease and sgRNA to *Trp53* gene (Adeno-sgp53-Cas9; Viraquest, North Liberty, IA, USA) followed by intramuscular injection of the carcinogen 3-methylcholenthrene (MCA). Transplant sarcomas were induced by intramuscular injection of 10^5^ cells in 100 µL sterile PBS into the gastrocnemius muscle of *Rag2*^−/−^ (Jackson Laboratory, Bar Harbor, ME, USA, stock #008449) and littermate control *Rag2*^+/−^ mice. *Rag2*^−/−^ mice do not produce mature T cells or B cells and therefore served as a model to generate lymphocyte-deficient tumors [19,22]. By contrast, *Rag2*^+/−^ mice have mature B and T cells and therefore were used to generate tumors with lymphocytes. 

### 2.2. Animal Experiments and Data Processing

In vivo micro-CT studies using both EID and PCD-based imaging were performed in *Rag2*^−/−^ (n = 12) and *Rag2*^+/−^ (n = 13) mice. When tumors reached 60–70 mm^3^, they were irradiated with 20 Gy in a single fraction using the Precision Xrad 225Cx small animal image-guided irradiator [23]. Immediately after radiation, mice were intravenously injected with liposomal-iodine contrast agent (Lip-I, 0.012 mL/g mouse) via retro-orbital route. Lip-I was fabricated using methods described previously [15]. We have previously reported on using liposomal NPs for vascular and tumor imaging using spectral PCD-based CT imaging [13]. 

#### 2.2.1. In Vivo Micro-CT Imaging

Five days after NP contrast injection, mice were imaged under isoflurane anesthesia (2%) with our unique dual source hybrid spectral micro-CT system, acquiring data with both the EID and a PCD imaging chain [13]. The system is equipped with two Varian G297 X-ray tubes and the Dectris Santis 1604 PCD. This PCD has four energy bins, 150 µm pixels and a 1 mm CdTe sensor. The scanner is set up such that the subject (e.g., a mouse) is mounted vertically in a 3D-printed cradle made of polylactic acid and rotated through all projection angles. The exposure settings for the PCD CT scan were 80 kVp with Cu filtration (0.1 mm), 2 mA, 200 ms per projection. The PCD energy thresholds are set to 25, 34, 50 and 60 keV. The first threshold (25 keV) is selected to limit spectral distortions due to charge sharing and to remain well above the detector’s noise floor (<6 keV). The next two thresholds are chosen in proximity of the K-edge of iodine (33.2 keV). The last threshold at 60 keV is selected to provide sufficient photon counts in the last energy bin. A total of 900 views were acquired over 1070° rotation. To extend the PCD field-of-view along the *z*-axis and to minimize ring artifacts in our reconstructions, scans are performed using a helical trajectory with 1.25 cm vertical translation during acquisition. For the EID CT scan, the following scanning parameters were used: 50 kVp with 0.1 mm Cu filtration, 80 mA and pulsed for 12.5 ms per projection (720 projections). Sampling for both chains required 3 min. We have previously reported on both simulations and physical phantom experiments to validate the optimal scanning kVps for DE-CT preclinical imaging of I-based contrast agents [24]. For the EID chain, maximum contrast discrimination for I was found at 50 kVp with Cu filtration (0.1 mm). The radiation dose was 36 mGy for the EID scan and 50 mGy for the PCD scan. 

After imaging, mice were euthanized, and the tumors harvested for analysis by immunohistochemistry or flow cytometry. Immunohistochemistry was performed on paraffin-embedded tumor sections. Tissue specimens were fixed in 10% neutralized formalin for 24 h and preserved in 70% ethanol prior to paraffin embedding. Four micron sections were de-waxed with xylene and rehydrated with a graded series of ethanol and water washes prior to staining. For immunohistochemical staining of macrophages, the primary antibody utilized was a monoclonal rat anti-mouse F4/80 IgG2b (1:100, Bio-Rad, Hercules, CA, USA, clone A3-1, # MCA497), and the secondary antibody utilized was goat anti-rat IgG antibody conjugated to HRP (1:100, Invitrogen, Waltham, MA, USA, #18-4818-82). Quantification of F4/80 staining was performed by an observer blinded to genotype using the IHC toolbox plugin available in Image J (https://imagej.nih.gov/, accessed on 1 November 2021).

For flow cytometry, tumors were dissected and minced before dissociation per manufacturer’s instructions for tough murine tumors using MACS C tubes and the mouse Tumor Dissociation Kit (Miltenyi Biotec, Bergisch Gladbach, Germany). After the tumor dissociation was completed, the cells were filtered through a 40 μm strainer. RBCs were lysed using ACK Lysing Buffer (Lonza, Basel, Switzerland) and washed with flow buffer made of HBSS (cat 13175-095, Gibco, Waltham, MA, USA), 5 mM EDTA (E7889, Sigma-Aldrich, St. Louis, MO, USA), and 2.5% fetal bovine serum (FBS; Gibco, Waltham, MA, USA). The cells were blocked with purified rat anti-mouse CD16/CD32 (Clone 93; Biolegend, San Diego, CA, USA, #101320) then stained with APC-Cy7 rat anti-mouse CD45 antibody (BD Biosciences, Franklin Lakes, NJ, USA, 557659) and BV711 hamster anti-mouse CD3e antibody (BD Biosciences, Franklin Lakes, NJ, USA, 563123) for 25 min on ice. After washing, cells were stained with Live/Dead Zombie Aqua (Biolegend, San Diego, CA, USA, 1:500) for 15 min. After washing, the cells were fixed in 1% paraformaldehyde (ThermoFisher Scientific, Waltham, MA, USA). Flow cytometry was performed in the Duke Human Vaccine Institute Flow Cytometry Facility (Durham, NC, USA) using the BD LSR II cytometer. Quantification of live CD3+ cells was performed by an observer blinded to genotype.

#### 2.2.2. Image Reconstruction

**Figure 2:**
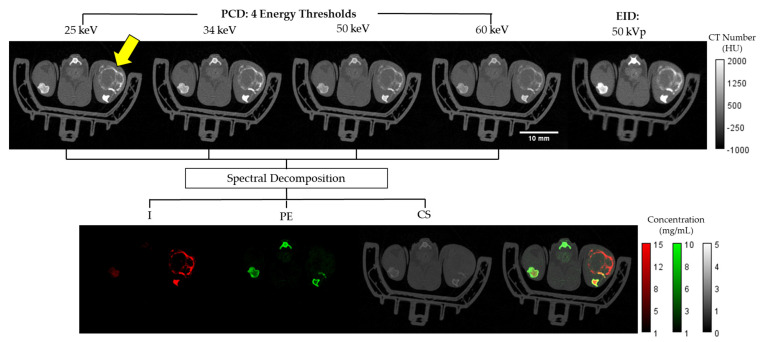
Axial slices from a *Rag2*^+/−^ mouse. Shown at the top row are Hounsfield unit (HU)-normalized images from the four energy bins for the PCD and the EID CT. The bottom row shows the three material color-coded maps in mg/mL (I, PE, CS) as well as a composite representation of the three materials overlaid. The tumor region is identified by the yellow arrow within the upper left PCD image.

The EID and spectral PCD CT data were registered and independently reconstructed with the same isotropic voxel size of 125 µm using the iterative spectral CT reconstruction framework that we have described previously [25,26]. Specifically, we applied the split Bregman method with the add-residual-back strategy [27] and rank sparse kernel regression regularization [28]. The top row of Figure 2 shows an example axial slice from the reconstructed multi-energy PCD and EID CT data.

#### 2.2.3. Material Decomposition

To decompose spectral CT data into material maps, we used a post-reconstruction decomposition method [13] with PE, CS, and I as material basis functions. Vials of known concentrations of I, Ca and water were used to construct a sensitivity matrix. Spectral decomposition was performed by solving the following linear system at each voxel:

(1) $$x=A^{-1}b$$

A is a sensitivity matrix measured for I, PE, and CS at each energy $E_{i}$. Finally, b is the intensity of the voxel under consideration at $E_{i}\;\mathrm{with}\;i=1\ldots 4$ where the four energies correspond to the four PCD energy thresholds. As mentioned above, these thresholds were set at 25 keV, 34 keV, 50 keV, and 60 keV to optimize for the k-edge of Iodine and minimize noise. The bottom panel of Figure 2 shows a representative example of the I, PE, CS decompositions and their combined color-coded display.

#### 2.2.4. Tumor Segmentation

Using ITK-SNAP (http://www.itksnap.org, accessed on 6 September 2021), we segmented the tumors in the lowest energy PCD-CT image. Due to the registration of the EID and PCD CT imaging chains, the same segmentation masks applied for all CT sets corresponding to the same animal. The segmentation was performed using a semi-automated, seed-based, region growing approach verified by qualitative inspection. The tumor boundaries were not always clearly marked by visible contrast differences, but care was taken to be as consistent as possible across all animal subjects.

### 2.3. Radiomic Analysis

#### 2.3.1. Semantic Radiomic Feature Calculation

The segmentation masks were used to measure a selection of tumor metrics relating to iodine contrast enhancement. Iodine concentrations less than 1 mg/mL within the tumor mask were discarded. This threshold of 1 mg/mL was selected due to the limits of detection and the presence of high levels of noise at low concentrations [13]. With this iodine information and the segmentations, we quantified tumor volume, iodine enhanced tumor volume, the corresponding enhanced tumor percent, the accumulated iodine mass, and the mean iodine concentration. These conventional metrics are termed “semantic” radiomic features. They are quantitative features which have been calculated using radiological images; however, we draw a distinction between these quantitative measures that are traditionally interpretable by a radiologist and the “agnostic” radiomic features which include the more complex quantitative measures such as texture and shape typically associated with the field of radiomics [5]. These semantic features serve as a comparative baseline when analyzing the effectiveness of subsequent radiomic analysis using more complex agnostic features.

#### 2.3.2. Agnostic Radiomic Feature Calculation

Following this baseline quantitative image analysis based on semantic radiomic features, we calculated a wide array of agnostic radiomic features (RFs) from the segmented tumor region. RFs were calculated using the open source PyRadiomics package [29] and included features from the following families: morphology, first-order statistics, first-order histogram, and second-order texture (i.e., gray level co-occurrence matrix, gray level run length matrix, gray level size zone matrix, neighboring gray tone difference matrix, and gray level dependence matrix). Histogram and texture RFs were calculated using the original form of the images as well as after a wavelet decomposition. In total, 851 agnostic RFs were extracted from each 3D tumor volume independently for the different imaging modalities. In all, the number of calculated RFs were 851 for the EID images, 3404 for the PCD images, and 2553 for the material map. In our analysis, we had a total of eight volumes corresponding to EID data, four energy bins of our PCD data, and three decomposed material maps (I, PE, CS). We chose to group our RFs into three groups based on data type (EID, PCD, material maps) to explore and compare relative diagnostic performance between the different imaging methodologies.

#### 2.3.3. Univariate Radiomic Analysis

To assess the potential association between each of the individual features and the *Rag2* genotype, we performed unpaired Wilcoxon-Mann-Whitney tests on each of the features. Statistical significance was determined with a Benjamini–Hochberg multiple hypothesis correction with a significance level of 0.05 [30].

#### 2.3.4. Multivariate Radiomic Analysis

To select significant RF subsets and remove redundant or irrelevant features, we used the Maximum Relevance Minimum Redundancy (MRMR) algorithm. This algorithm seeks to find a statistically relevant subset of features while also penalizing for redundancy [31]. To decide how many features to select, we first performed a principal component analysis (PCA) to find the number of singular vectors that captured 90% of the variability of each feature space as described previously [32]. This number is an estimation of the optimal dimension to be used for an RF subset and the subsequent downstream machine learning models.

As part of our multivariate analysis, we trained a logistic regression binary classifier for each of the RF subsets (i.e., EID, PCD, material maps). We used a stratified Monte Carlo repeated cross validation strategy to assess classifier performance throughout the training process [32]. With this approach we took 50 different samples of five randomly subsampled validation folds. Stratification was performed verifying that each fold’s validation subsets had at least two of each genotype to maintain relatively equal class representation. Within each of the folds, we first used the MRMR algorithm to choose the top features for that specific subset of mice. A logistic regression model was then fit to the training subset and validation tests were performed. Classification performance metrics were calculated for each of the 50 repeated subsamples. The model predictions were compiled into receiver operating characteristic (ROC) curves. Performance was quantified using the area under the ROC curve (AUC), precision, recall, and prediction accuracy. Models corresponding to the EID images, PCD images, and material maps were trained in parallel to compare their relative performance. Non-parametric Wilcoxon Signed Rank tests were used to perform pair-wise statistical comparisons between the AUC values of the three models [32,33,34].

The upper limit of how many RFs for the MRMR algorithm to choose was determined by our preliminary PCA analysis. We then performed an exhaustive search to observe how the classifier performance varied based on the number of features chosen ranging from 1 to the upper limit. This search then informed our final choice of feature numbers. 

## 3. Results

### 3.1. Analysis of Semantic Radiomic Features

**Figure 3:**
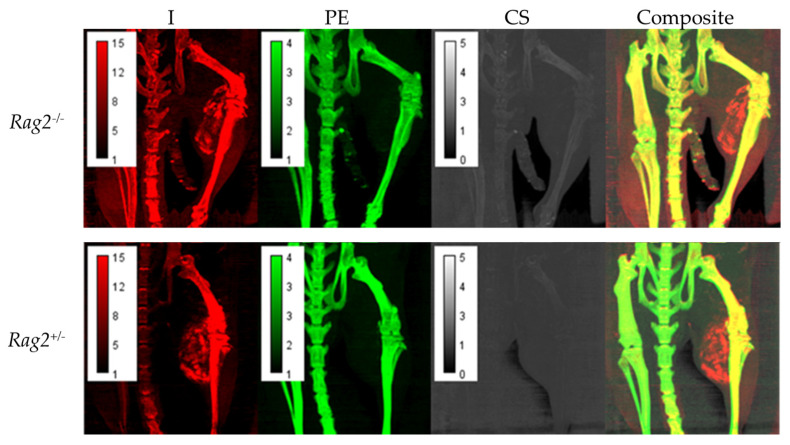
Maximum intensity projections (MIPs) of the decomposition maps for *Rag2*^−/−^ and *Rag2*^+/−^ mouse demonstrating intratumoral nanoparticle accumulation and iodine enhancement as well as the overall quality of the decomposition results. Each pixel in these images represents the maximum voxel value across a projection through a given number of slices. In this case, we have chosen 20 coronal slices to capture the tumor vasculature.

Figure 3 displays maximum intensity projections (MIP) of the decomposition maps for a *Rag2*^−/−^ and *Rag2*^+/−^ mouse demonstrating heterogeneous intratumoral Lip-I accumulation and iodine enhancement as well as overall quality of the decomposition results.

**Figure 4:**
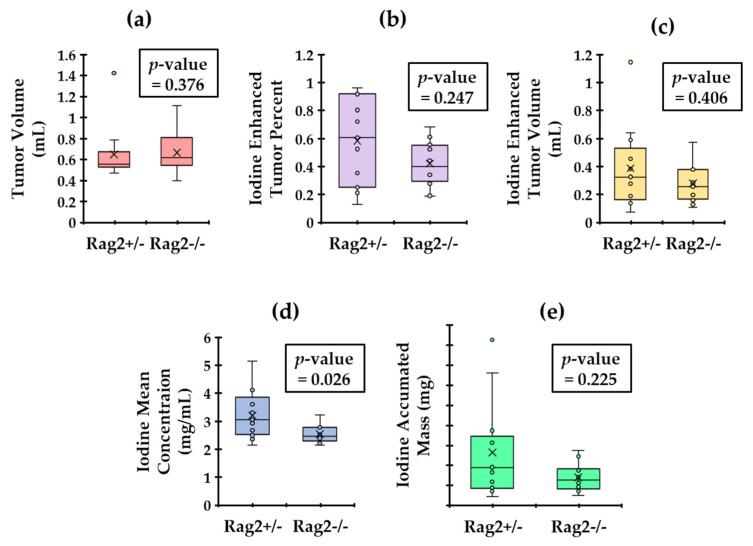
Calculated tumor metrics with the associated p-value from an unpaired Wilcoxon–Mann–Whitney test. We display the comparison of tumor volume (**a**). We also show plots of the Iodine enhanced tumor volume (**b**), the enhanced tumor percentage (**c**), the mean iodine concentration (**d**) and the iodine accumulated mass (**e**) in tumors. Only the mean iodine concentrations are significantly different between the two genotypes (*p* < 0.05).

The results of our semantic RF analysis calculated using the decomposed iodine maps are reported in the box plots of Figure 4. The *p*-values for Wilcoxon–Mann–Whitney test are also shown within each plot. The results show that mean iodine concentration in the tumors is the only statistically significant (*p* < 0.05) distinguishing semantic RF for these two groups. This suggests that the *Rag2*^+/−^ mice (that have lymphocytes) have leakier tumors than the *Rag2*^−/−^ mice. 

### 3.2. Analysis of Agnostic Radiomic Features

#### 3.2.1. Univariate Analysis

Our univariate statistical analysis with multiple hypothesis corrections revealed that 12 of the EID RFs, 24 of the PCD RFs, and 20 of the material decomposition RFs were statistically significant. 

#### 3.2.2. Multivariate Analysis

**Figure 5:**
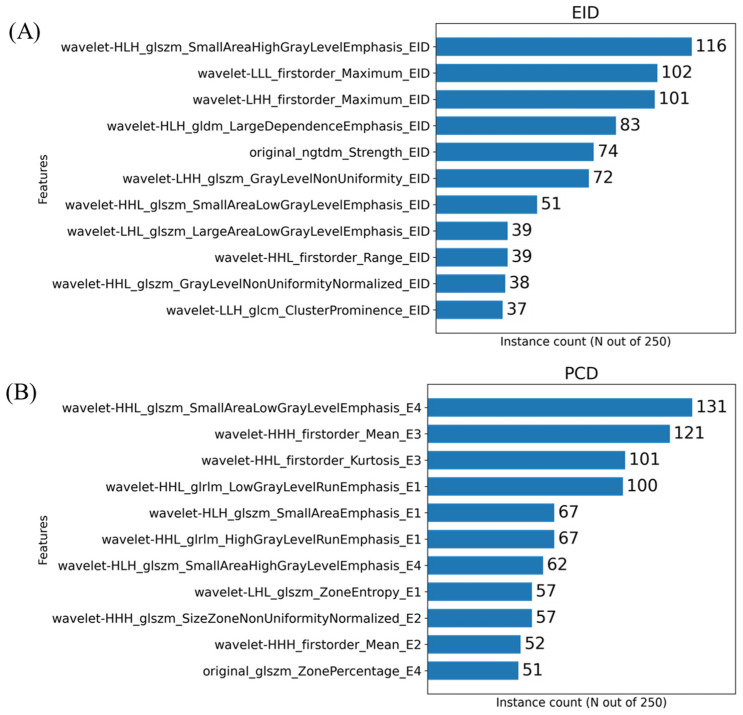

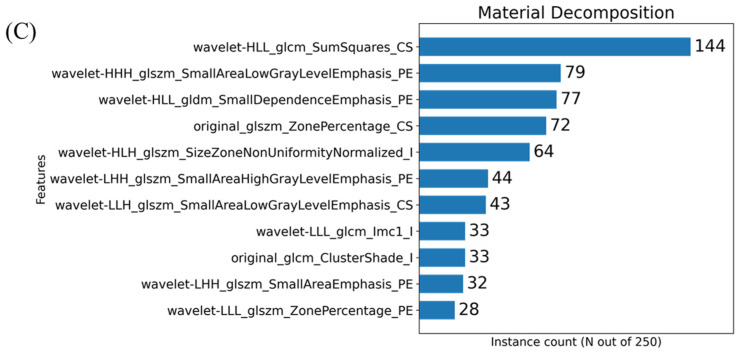
Histograms resulting from the MRMR-based feature selection process for the three imaging sets i.e., EID (**A**), PCD (**B**) and material decomposition (**C**). The histograms show the frequency of a given RF selection throughout the repeated cross validation folds. For example, a feature that was found to be significant for all 5-folds across all 50 repeated samples would have a value of 250. The feature names are written in a form output from PyRadiomics with an appended suffix used for identifying the image source. Each name is the union of the following: image transform, feature class, feature name, image type. For example, the RF representing the calculated 3-dimensional sphericity from the raw EID images, with no wavelet decomposition, would be written as “original_shape_Sphericity_EID”. Only the top 11 most repeated features are shown on these plots for readability.

**Figure 6:**
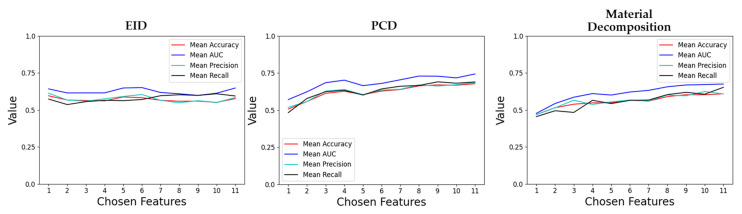
Plots of various performance metrics as functions of the number of features chosen for classifier training. One plot is shown for each of the RF types (EID, PCD, material decomposition). These plots represent the results of an exhaustive search analyzing how classification performance varies when choosing different numbers of features ranging from 1 to 11.

The histograms presented in Figure 5 show the number of times a given feature was chosen by the MRMR algorithm across our cross-validation folds. In the case of these plots, 11 features were chosen within each fold. Our initial PCA analysis revealed that approximately 11 components were needed to capture 90% of the variability of the various feature sets. Figure 6 shows the effect that the number of features selected has on classification performance for the three feature sets. Based on the trends identified in Figure 6, we chose to use 11 features when training our classifiers to achieve the peak performance.

**Figure 7:**
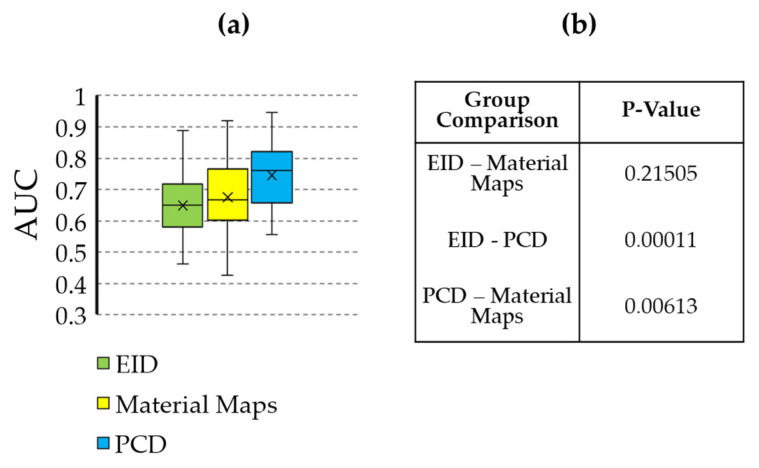
Statistical comparison of the AUC values resulting from classification training using the different feature sources (EID, PCD, material maps). (**a**) Box and whisker plot showing the AUC values. (**b**) Table containing the *p*-values comparing each grouping. The AUC of the PCD classifier is significantly higher than that of the EID and material maps.

**Figure 8:**
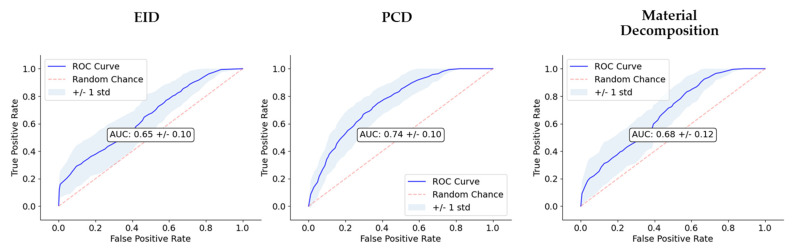
Comparison of classifier performance. We show the receiver operating characteristic (ROC) curves for each of the three image types (EID, PCD, material maps). In the center of the plots, AUC is also reported. The dashed line along the diagonal of the ROC is included as a reference to show how the ROC of a random, no-skill classifier would appear.

The performance of the classifiers trained on the three RF sets are summarized in Figure 7 and Figure 8, and Table 1. Model performance was maximized for the PCD RF set, which yielded an AUC of 0.74 and an accuracy of 0.68.

**Figure 9:**
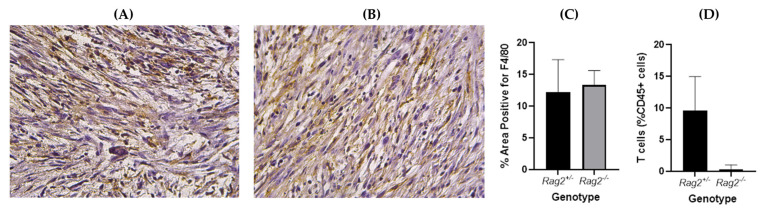
Representative immunohistochemistry slides of sarcoma tumors in (**A**) *Rag2*^−/−^, (**B**) *Rag2*^+/−^ mice stained with F4/80 (macrophage marker). The slides were analyzed and the percentage area positive for F4/80 was found not to be significantly different between the two genotypes (*p* = 0.62, unpaired *t*-test) (**C**). Flow cytometry confirmed significantly higher T cell infiltrate in tumors from *Rag2*^+/−^ compared to *Rag2*^−/−^ mice (*p* = 0.01, unpaired *t*-test) (**D**).

Tumors in mice from the two genotypes show similar macrophage levels (Figure 9) as indicated by immunohistochemistry with F4/80 staining. A significant difference in T cell infiltrate between the genotypes was confirmed by flow cytometry. 

## 4. Discussion

Our radiomic analysis is based on the use of a Lip-I NP contrast agent for CT imaging. Unlike conventional contrast agents which have rapid wash-in/wash-out kinetics in tumors, NPs extravasate and reside in tumors for a prolonged period due to EPR phenomenon associated with abnormal tumor vasculature [35]. Thus, delayed CT imaging of NP can benefit radiomic analysis, since it eliminates artificial variations in signal enhancement patterns associated with rapid kinetics of the contrast agent, a phenomenon commonly observed with conventional low molecular weight iodine-based imaging agents. The same NP contrast agent, i.e., Lip-I, was used in combination with high-resolution CT imaging to probe the tumor microenvironment in models of varying TAM burden [20]. Previous studies have also shown its use in probing tumor vasculature for differentiation of other tumor phenotypes and monitoring tumor response to vascular-targeted therapies [14,36].

With the initial baseline quantitative image analysis, the mean iodine concentration was found to be statistically significantly different between the two groups (Figure 4). The *Rag2*^+/−^ mice (that have lymphocytes) demonstrated a higher mean iodine concentration relative to the *Rag2*^−/−^ mice. A potential explanation links to the fact that lymphocytes can release cytokines and chemokines that contribute to leaky vasculature [37], thus leading to higher NP accumulation and therefore high iodine concentration in tumors of *Rag2*^+/−^ mice. Thus, this baseline analysis informed us of a tangible phenotypical difference between the two genotypes. Figure 9 eliminates the hypothesis that changes associated with radiomics may be attributed to other causes such as the macrophage burden.

However, radiomic analysis facilitates characterization of complex radiogenomic interactions that can be used to study basic biology and identify prognostic markers during treatment [38,39]. Agnostic features have been shown to add complementary insight compared to conventional semantic features, including disease staging and tumor volume [40]. Based on the most commonly selected radiomic features (Figure 5), the texture and shape-based features found from the image wavelet transforms were most informative. Our multivariate classification models demonstrated a peak accuracy of 0.68. This peak accuracy was associated with the model derived from PCD-based RFs. The PCD-CT based performance was superior both to that of the models of material map RFs (0.60) and that of the models from EID-derived RFs (0.58). The results in Table 1 and Figure 7 and Figure 8 motivate the superiority of the PCD CT compared to EID CT. Thus, we have shown that the use of PCD CT data increases the accuracy and effectiveness of radiomic analysis beyond what typical single-energy EID RFs are able to provide. However, the decreased classification accuracy using the RFs from the derived material decompositions suggests that additional abstractions of the data, in this case performing a material decomposition on PCD data, can amplify noise and remove potentially useful information. These results remain limited due to the small sample size of our data, and thus warrant future investigation and follow up experiments. Nevertheless, our results support the continued development of machine learning based tools in radiomics as a standard approach with the performance they have shown in a growing array of high demand radiological applications.

Although the use of PCD CT is now being evaluated on some clinical research prototypes [41], preclinical imaging still represents an excellent development and testing environment for spectral PCD CT technologies with potential for clinical translation. Many groups are developing new prototype PCD micro-CT systems and new contrast agents to explore their capabilities at preclinical levels. Currently, there is one commercially available PCD-based micro-CT system, the Medipix All Resolution System (MARS Bioimaging Ltd.; Christchurch, New Zealand) [42]. The MARS scanner uses the Medipix3 detector chip developed at CERN (Geneva, Switzerland) with charge-summing circuitry to compensate for charge sharing between neighboring detector pixels [43].

A limitation of our work is the small sample size, which was limited due to the complexity of our experimental design. Because of this, our current results do not yield a final set of radiomic features to be independently tested. Future work will focus on larger sample sizes to be used as an independent testing set on which to estimate the true generalization capacity of our models. Still, the findings reported in this manuscript provide novel hypothesis generating data, which are essential to designing future pre-clinical radiogenomic studies. Another limitation of the present study is the lack of data in mice without radiation. Such control groups will help to verify the intrinsic differences in our genetically engineered mice.

In conclusion, this study shows that tumors with different lymphocyte burden manifest subtle changes in tumor architecture that are not detected with imaging-derived conventional tumor metrics but are better revealed on radiomic analysis of nanoparticle contrast-enhanced PCD CT images. With the clinical promise of radiomics coupled with the emerging clinical use of PCDs, these findings exemplify the benefits of these technologies. 

## Figures and Tables

**Table 1:** Validation test results from classification training for each of the different feature sets. The decision thresholds used in the calculation of accuracy, precision, and recall correspond to the average intersection point of precision and recall within each repeated 5-fold.

Feature Source	Accuracy	Precision	Recall	AUC
EID	0.58 ± 0.08	0.59 ± 0.10	0.58 ± 0.14	0.65 ± 0.10
PCD	0.68 ± 0.09	0.69 ± 0.11	0.68 ± 0.12	0.74 ± 0.10
Material Maps	0.60 ± 0.10	0.61 ± 0.12	0.60 ± 0.14	0.68 ± 0.12

## Data Availability

The Radiomic feature data as well as the code used for training and analysis can be found at https://gitlab.oit.duke.edu/aja54/rt-radiomics (accessed on 16 December 2021).
